# Clinical Significance of Circulating Tumor Cells (CTCs) and Survivin on Predicting Prognosis in Thyroid Cancer Patients

**DOI:** 10.1155/2022/5188006

**Published:** 2022-01-31

**Authors:** Xun Weng, YangYang, Yujiao Cai

**Affiliations:** Department of General Surgery, Xinqiao Hospital, Army Medical University, No. 83 Xinqiao Main Street, Shapingba District, Chongqing 400037, China

## Abstract

**Background:**

Clinical significance of circulating tumor cell (CTC) count, mesenchymal CTCs (MCTCs), and survivin in patients with thyroid cancer remains unclear. We evaluated the relationship between the expression of different CTC subtypes or survivin and the prognosis in patients with thyroid cancer. *Patients and Methods*. This study enrolled 164 patients with thyroid cancer who were diagnosed from January 2013 to September 2020 in our hospital. Among these patients, there were 73 cases with papillary thyroid cancer (PTC), 60 cases with follicular thyroid cancer (FTC), 12 medullary thyroid cancers (MTC), 10 poorly differentiated thyroid cancers (PDTC), 9 anaplastic thyroid cancers, and 10 control patients with nonmalignant thyroid nodules based on their histopathological characteristics. Only 5 milliliters (mL) of peripheral blood from the patients with thyroid cancer and control was used to detect the CTC cell number via CanPatrol capture technique before treatments. We also isolated mononuclear cells (MNC) from the peripheral blood and performed quantity reverse transcriptase polymerase chain reaction (qPCR) for survivin gene expression among these patients.

**Results:**

The overall positive rates of CTC at diagnosis were 56.1%. The relapse and metastasis rates in PTC and FTC patients with more than 6 CTCs and positive MCTCs were significantly higher than those in the patients with 6 or less than 6 CTCs and MCTCs. It was also found that these patients with >6 CTCs and MCTCs had shorter progression-free survival (PFS). Additionally, the survivin level of the patients with thyroid cancer was strongly relative to differentiation grades of thyroid cancers.

**Conclusions:**

The detection of more than six of total CTCs and positive MCTCs in the patients with differentiated thyroid cancer is an excellent biomarker for predicting the prognosis of patients. Survivin also is a good biomarker for thyroid cancer differentiation.

## 1. Introduction

Worldwide, palpable thyroid nodules can be detected in around 5% of women and 1% of men [1]. Among these patients, only 7-15% patients are malignant [2]. Initial screening of patients with thyroid nodules is currently to utilize ultrasound and fine needle aspiration biopsy (FNAB) [3]. The differentiation between benign and malignant thyroid nodules is still determined by histological examination from the excised thyroid tumor via nodule biopsy or FNAB [4]. Therefore, a few methods, including specific gene detection by polymerase chain reaction (PCR) technique, have been developed for differentiating from benign and malignant thyroid nodules preoperatively [5]. According to the World Health Organization (WHO) on the latest classification of thyroid cancer in 2017 [6], thyroid cancer is mainly classified into papillary thyroid carcinoma (PTC), follicular thyroid carcinoma (FTC), Hürthle (oncocytic) cell carcinoma (HCC), medullary thyroid carcinoma (MTC), poorly differentiated thyroid carcinoma (PDTC), and anaplastic thyroid carcinoma based on their histopathological characteristics [7, 8]. Among these major classifications, they can further divide into different subclassifications based on their molecular and histopathological feathers [9, 10]. The treatments of the patients with thyroid cancer include surgery, radiation therapy, chemotherapy, hormone therapy, and targeting therapy [11–13]. Surgical resection no doubt is the most effective therapy tool for nonmetastatic thyroid cancer. The prognosis of thyroid cancer is relevant to its histopathological type. Normally, the overall prognosis of PTC and FTC is excellent. Their 5-year overall survival (OS) rate is around 96% [14]. However, HCC, MTC, PDTC, and anaplastic thyroid carcinoma at advanced stages III-IV have very poor prognosis [15]. Therefore, it is urgent to find more reliable and accurate biomarkers for predicting prognosis of patients with thyroid cancer.

Recent studies showed that circulating tumor cells (CTCs) were originally cells from the primary tumor and can be released into bloodstream, which then give rise to tumor metastasis [16, 17]. Different resources have demonstrated that CTC count is a sensitive biomarker to predict tumor progression and helps to make the decision for treatments [18–20]. For example, a study showed that higher CTC level in patients with hepatocellular cancer had strong correlation with early relapse [21]. The other report revealed that epithelial cells from primary tumors can enter into adjacent tissues by the epithelial-mesenchymal transition (EMT) mechanism [22]. CTCs are classified into epithelial type, mesenchymal CTC (MCTC) type, and both mixed types according to their surface markers [23]. So far, CTC evaluation for thyroid cancer mostly has only a few available data, and the results revealed a little correlation with CTC number [5, 23, 24]. Here, as in the previous description, PTC and FTC have good 5-year OS. In contrast, MTC, PDTC, and anaplastic thyroid carcinoma have poor outcomes. Therefore, our study focused on the relationship between CTCs and PTC or FTC patients.

Previous studies showed that thyroid-specific genes, including *SLC5A5*, *TG*, *TPO*, and *TSHR*, may be excellent candidates for thyroid cancer development [25]. However, some biomarkers like *TSHR* specificity have been questioned [26]. Therefore, more new biomarkers need to be validated. Recent studies revealed that survivin, a member of the inhibitor of apoptosis (IAP) family [27], is a good biomarker for distinguish benign and malignant thyroid nodules because it is expressed in most cancer cells, not in normal tissues [28–30]. In addition, survivin is also an excellent target for cancer therapy because of its absence in normal cells [31]. In the current study, we detected survivin gene expression and CTC levels of blood circulation from patients with thyroid cancer. This study was to evaluate the clinical significance of CTCs and survivin in the patients with thyroid cancer.

## 2. Materials and Methods

### 2.1. Patient Samples

We retrospectively analyzed 164 patients, including PTC, FTC, MTC, PDTC, and anaplastic thyroid carcinoma corresponding to 73, 60, 12, 10, and 9 cases, respectively. These patients were diagnosed based on their histopathological characteristics in our hospital from January 2013 to September 2020. The samples of 10 patients with benign thyroid nodules were the negative control. Diagnosis and staging criteria of all patients were based on the 8^th^ edition of the American Joint Committee on Cancer (AJCC) staging system for thyroid cancer [32]. All cells were from the peripheral blood of patients at diagnosis. All patients were followed up every 5 months after treatment. For patients with possible recurrence, they were followed up every 2 months. Progression-free survival (PFS) was calculated from the time of the first treatment to relapse or distant metastasis. This study protocol was reviewed and approved by the hospital ethics committee of Xinqiao Hospital, Army Medical University. Written informed consent was obtained from all participated patients.

### 2.2. Detection of CTCs via CanPatrol and Tricolor RNA-ISH Methods

**Figure 1:**
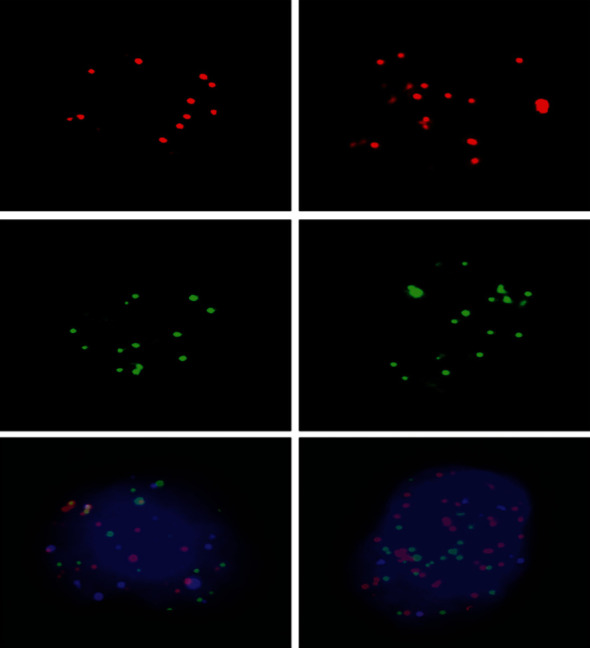
Images of CTCs. Upper, middle, and lower panels show the pictures of epithelial, mesenchymal, and mixed CTCs, respectively. Epithelial CTCs were stained with only Alexa Fluor 594-labeled epithelial markers EpCAM and CK8/18/19. Mesenchymal CTCs were stained with Alexa Fluor 488-labeled Vimentin and Twist. Pictures were taken with 40x magnification with a immunofluorescence microscope.

The characteristic strategies of CTC in patients with thyroid cancer were followed as per the previous description [33]. Briefly, 5 mL peripheral blood was taken from the patients as well as the control at diagnosis. It was spun for 5 minutes at 1500 revolutions per minute (rpm) within four hours after harvesting. The upper plasma phase was discarded, and CTCs were isolated via use of the CanPatrol CTC enrichment technique (SurExam, Guangzhou, China). Alexa Fluor 594-labeled epithelial makers (EpCAM, CK8/18/19), Alexa Fluor 488-conjugated mesenchymal markers (Vimentin and Twist), and nuclear marker (DAPI) were applied to identify CTCs by a tricolor RNA in situ hybridization technique [7]. CTCs were divided into epithelial, mesenchymal, and both mixed types according to the combination of their surface markers with DAPI (shown in Figure 1).

### 2.3. Survivin Expression by Quantity Reverse Transcription Polymerase Chain Reaction (qPCR)

A peripheral blood mononuclear cell (PBMC) was isolated from 5 mL whole blood of patients before treatment, and 1 mL of TRIzol reagents was added. Total RNA and cDNA syntheses were taken by commercial reagents. Survivin and internal control gene GAPDH TaqMan probe was purchased from Invitrogen. Survivin expression was calculated by *ΔΔ*Ct normalized GAPDH.

### 2.4. Statistical Analysis

The relationship between the CTC-positive rate and the clinic-pathological characteristics was counted via the *χ*^2^ test. Progression-free survival (PFS) was calculated from the time from initial treatment to local recurrence or distant metastasis by the Kaplan–Meier method and the log-rank test. All results were counted using the GraphPad Prism 9 software. When two-tail *P* values were <0.05, this was considered as statistically significant.

## 3. Results

### 3.1. Clinical Characteristics

This study enrolled 164 patients with thyroid cancer at TI-V tumor, node, metastasis (TNM) staging as well as 10 negative controls. The clinic-pathological features of patients are shown in Table 1.Their clinical parameters included age, sex, histology, and TNM staging. The most patients were diagnosed as differentiated thyroid cancer like 73 PTC (44.5%, 71/164) and 60 FTC (36.6%, 60/164). In addition, we also recruited poor differentiated thyroid cancer-like 12 MTC (7.3%, 12/164), 10 PDTC (6.1%, 10/164), and 9 ATC patients (5.5%, 9/164%) for this study. The peripheral blood from these patients was taken for CTC detection and survivin gene expression measurement.

### 3.2. Identification of CTC Subtypes in Patients with Thyroid Cancer

Samples of 164 thyroid cancer patients and 10 healthy controls were used to enrich CTCs by the CanPatrol technique. CTCs were classified into epithelial, mesenchymal, and both mixed subtypes based on their surface markers with different immunofluorescence dye staining (Figure 1). Our data revealed that the most patients only had a kind of CTC. Only a few epithelial CTCs were detected in benign control. It was found that there were CTCs in 92 out of 164 thyroid cancer patients (56.1%). We compared the CTC-positive rate based on age, gender, pathological subtypes, and TNM staging of the patients with thyroid cancer. It was identified that the positive rate of CTCs was not a positive correlation with age and gender of the patients (*P* > 0.05). In contrast, CTC-positive rate in different pathological subtypes and TNM staging had dramatic differences. The undifferentiated thyroid cancer, including MTC, PDTC, and ATC patients, and III-IV TNM stages had markedly high CTC-positive rate (*P* < 0.001) (Table 1).

### 3.3. Comparison of CTC Subtypes in the Patients with Differentiated Thyroid Cancer

**Figure 2:**
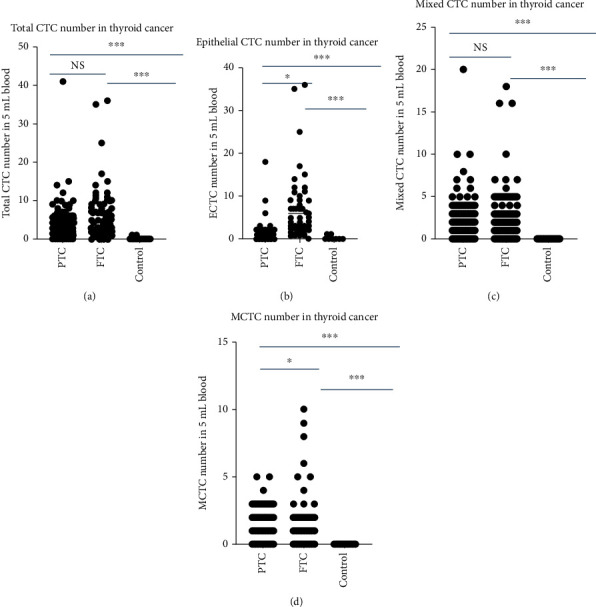
Comparison of CTCs and subtype CTC in PTC, FTC, and control patients: (a) total CTC number comparison; (b) epithelial CTC number comparison; (c) mixed CTC number comparison; (d) MCTC number comparison. PTC: papillary thyroid cancer; FTC: follicular thyroid cancer; CTCs: circulating tumor cells; MCTC: mesenchymal circulating tumor cell; NS: no significant difference. ^∗∗∗^*P* < 0.001; ^∗^*P* < 0.05.

To assess the clinical significance of CTC number in different types of patients with thyroid cancer, we compared the total cell number of CTCs and CTC subtypes of major differentiated thyroid cancer PTC and FTC because MTC, PDTC, and ATC are undifferentiated thyroid cancer and have a short overall survival. The results are shown in Figure 2. For total CTCs and mixed CTCs, either PTC or FTC was dramatically higher than the control (Figure 2(a), *P* < 0.001). However, there are no significant differences between PTC and FTC (Figures 2(a) and 2(c)). In contrast, for epithelial CTCs (Figure 2(b)) and MCTCs (Figure 2(d)), although both PTC and FTC were dramatically higher than the control (*P* < 0.001), FTC was also markedly more than PTC (*P* < 0.05). These results indicated that epithelial CTCs and MCTCs in FTC were higher than PTC.

### 3.4. Prognostic Significance of CTC Counts and Subtypes before Treatment

**Figure 3:**
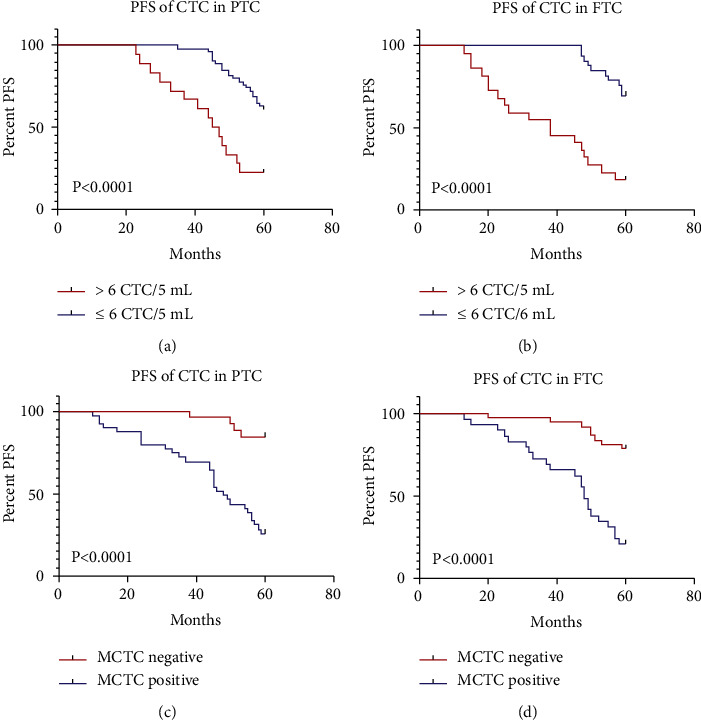
PFS of patients with CTCs and MCTC by Kaplan-Meier curves at diagnosis. (a) PFS in >6 CTCs vs. ≤6 CTCs of PTC patients; (b) PFS in >6 CTCs vs. ≤6 CTCs of FTC patients; (c, d) PFS comparison with MCTC vs. without MCTC in PTC and FTC patients. PTC: papillary thyroid cancer; FTC: follicular thyroid cancer; CTCs: circulating tumor cells; MCTC: mesenchymal circulating tumor cell.

To further evaluate the clinical significance of CTC subtypes, we followed up to 60 months for the patient prognosis. The results are shown in Figure 3 and Table 2. The analyses revealed that PFS in PTC patients (Figure 3(a)) or FTC patients (Figure 3(b)) with CTCs > 6 were significantly shorter (*P* < 0.001) than that in the patients with CTC ≤ 6 by the Kaplan–Meier survival curve analysis. In contrast, PFS of patients with positive MCTCs were also markedly shorter than those with negative MCTCs (*P* < 0.0001, Figures 3(c) and 3(d)). There was a huge difference among different groups (Table 2). Total CTC level > 6 in PTC or FTC had a big different hazard ratio and 95% confidence interval (CI) (PTC, HR 8.152, 95% CI 23.12-21.13, *P* < 0.0001; FTC, HR 5.531, 95% CI 2.33-13.03, *P* < 0.0001). Similarly, existence of MCTC (PTC, HR 0.131, 95% CI 0.07-0.026, *P* < 0.0001; FTC, HR 0.136, 95% CI 0.06-0.29, *P* < 0.0001) had dramatically poorer PFS.

### 3.5. Survivin Expression Is Significant Relevant to Thyroid Cancer Differentiation

**Figure 4:**
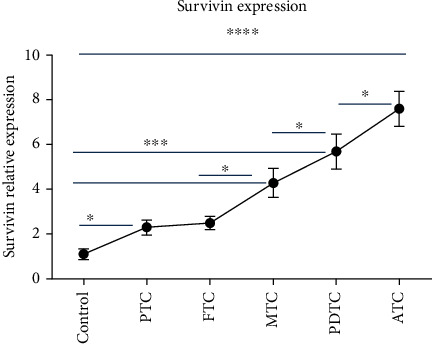
Survivin expression in thyroid cancer subtype. This data shows survivin relative expression by quantity reverse transcription polymerase chain reaction. PTC: papillary thyroid cancer; FTC: follicular thyroid cancer; MTC: medullary thyroid cancer; PDTC: poorly differentiated thyroid cancer; ATC: anaplastic thyroid cancer. ^∗∗∗^*P* < 0.001; ^∗^*P* < 0.05.

To investigate the relationship between survivin expression and thyroid cancer differentiation, we measured survivin gene expression by qPCR in patients with thyroid cancer subtype before therapy. The results are shown in Figure 4. With more poor differentiation, survivin had more high expression levels. ATC and PDTC had robust expression compared with the control (*P* < 0.001). In contrast, PTC or FTC only had some high expression (*P* < 0.05). Interestingly, survivin expression in ATC also was significant higher than PDTC (*P* < 0.05). In turn, survivin of PDTC was higher than that of PTC and FTC patients (*P* < 0.05). These results revealed that survivin is strong relative to the differentiated degree of thyroid cancer.

## 4. Discussion

Recent studies showed that CTCs were considered to be strongly relevant with cancer development [22, 34]. More data also revealed that CTC count of peripheral blood in cancer patients with advanced staging had an important guideline for predicting prognosis of the patients [18–20]. As for thyroid cancer, a few reports indicated that CTCs of the patients were involved in progress and prognosis [23, 24, 35]. However, CTCs of thyroid cancer patients at early stages have limited availability. Here, our results showed that total CTCs and their subtypes had a significant clinical association with the prediction of differentiated thyroid cancer prognosis.

CTCs in the bloodstream can be frequently characterized into epithelial, mesenchymal, and mixed types according to their surface markers with different immunofluorescence staining. Recent reports indicated that EMT-marker expressions in CTC were relevant to invasion and metastasis in many kinds of cancer like breast cancer, colorectal cancer, nonsmall cell lung cancer, gastric cancer, and prostate cancer [36–39]. For example, Bluemke et al. [40] found that positive CK+ CTCs in renal cancer patients were significantly relevant to their OS [41]. Xu et al. [24] study indicated that if there were more than 5 CTCs in patient's 5 mL peripheral blood, their prognosis were poor. In the present study, we selected two differentiated thyroid cancer PTC and FTC as our objects because their 5-year OS is long. So we can easily observe the relationships between CTC count and the outcomes. We found that if total CTCs and MCTCs of the PTC or FTC patients were high at diagnosis, they were most likely to have quick tumor progress. We also showed that if the patients had 6 total CTCs or positive MCTCs, their PFS were significantly shorter than the patients with less than 6 total CTCs or negative MCTCs. Similarly, patients with increased MCTC percentage after surgery relapse earlier in hepatocellular cancers [42]. Thus, monitoring of CTC subtypes and changes during treatment of thyroid cancer patients may provide another predictor of recurrence compared with conventional clinical parameters.

In addition to clinical significance of CTC, many studies also explored other biomarkers for the prognosis of thyroid cancer [43–45]. Among these biomarkers, survivin is an interesting gene because it is involved in many kinds of cancers [46, 47]. Survivin is an antiapoptosis protein and is expressed in most tumor cells, not in normal cells [27, 30]. Therefore, surviving is an excellent target for cancer therapy. The Wu et al. [29] study revealed that knockdown survivin may treat papillary thyroid cancer via targeting survivin. This result showed the high survivin promoted thyroid cancer proliferation. Indeed, our data indicated that higher survivin is strongly relevant to poor differentiated thyroid cancer. These data confirmed that survivin also is a good biomarker for the diagnosis and therapy of thyroid cancer.

## 5. Conclusion

The present study indicated that there was a relationship between CTC subtypes and PFS in patients with thyroid cancer. High CTCs or positive MCTCs were significantly correlated with early recurrence or metastasis. Survivin expression is positively associated with the differentiated degree of thyroid cancer.

## Figures and Tables

**Table 1:** Relationship between the presence of circulating tumor cells (CTCs) and the clinical features of thyroid cancer.

	Number of cases	CTC-positive (%)	CTC-negative (%)	*χ* ^2^	*P* values
Age					
≤60 years old	93 (56.7%)	54 (58.1%)	39 (42.9%)	1.72	0.46
**>**60 years old	71 (43.3%)	52 (73.3%)	19 (26.7%)		
Gender					
Female	104 (63.4%)	62 (59.6%)	12 (40.5%)	0.015	0.56
Male	60 (36.6%)	30 (50.0%)	30 (50.0%)		
Pathological subtypes					0.001
PTC	73 (44.5%)	12 (16.4%)	61 (83.6%)	10.63	
FTC	60 (36.6%)	14 (23.3%)	6 (76.7%)		
MTC	12 (7.3%	7 (58.3%)	5 (41.6%)		
PDTC	10 (6.1%)	9 (90.0%)	1 (10.0%)		
ATC	9 (5.5)	9 (100.0%)	0 (0.00%)		
TNM stages					
I	101 (61.4%)	9 (8.9%)	92 (91.1%)	10.63	0.001
II	30 (18.4%)	15 (50.0%)	15 (50.0%)		
III	22 (13.5%)	18 (81.8%)	4 (18.2%)		
IV	11 (6.7%)	11 (100.0%)	0 (0.00%)		

CTC: circulating tumor cell; PTC: papillary thyroid cancer; FTC: follicular thyroid cancer; MTC: medullary thyroid cancer; PDTC: poorly differentiated thyroid cancer; ATC: anaplastic thyroid cancer.

**Table 2:** Comparison of different CTC numbers on patient PFS.

Variables	HR	95% CI	*P* value
CTC in PTC > 6 vs. ≤6/5 mL	8.152	3.12 to 21.13	<0.0001
CTC in FTC > 6 vs. ≤6/5 mL	5.531	2.33 to 13.03	<0.0001
MCTC in PTC negative vs. positive	0.131	0.07 to 0.26	<0.0001
MCTC in FTC negative vs. positive	0.136	0.06 to 0.29	<0.0001

CTC: circulating tumor cell; PFS: progression-free survival; PTC: papillary thyroid cancer; FTC: follicular thyroid cancer; MCTC: mesenchymal circulating tumor cell; HR: hazard ratio; CI: confidence interval.

## Data Availability

The data used to support the findings of this study are available from the corresponding author upon request.
